# Mutations in VP1 and 5′-UTR affect enterovirus 71 virulence

**DOI:** 10.1038/s41598-018-25091-7

**Published:** 2018-04-27

**Authors:** Ching-Kun Chang, Shang-Rung Wu, Ying-Chin Chen, Kuen-Jin Lee, Nai-Hsiang Chung, Yi-Ju Lu, Shu-Ling Yu, Chia-Chyi Liu, Yen-Hung Chow

**Affiliations:** 1 National Institute of Infectious Disease and Vaccinology, National Health Research Institutes, Zhunan, 350 Taiwan; 2 Graduate Institute of Life Science, National Defense Medical Center, Taipei, 114 Taiwan; 3 Institute of Oral Medicine, National Cheng Kung University, Tainan, 701 Taiwan; 4 Graduate Program of Biotechnology in Medicine, Institute of Molecular and Cellular Biology, National Tsing Hua University, Hsinchu, 300 Taiwan; 5 Graduate Institute of Biomedical Sciences, China Medical University, Taichung, 404 Taiwan

## Abstract

Enterovirus 71 (EV71) is a major cause of hand, foot and mouth disease (HFMD). The current EV71 propagating in Vero (EV-V) or sub-passaged in RD (EV-R) cells was used as a pathogen. Interestingly, EV-R exhibited differential virulence; challenging human scavenger receptor class B2-expressing (hSCARB2-Tg) mice with EV71 revealed that EV-V was more virulent than EV-R: 100% of mice that received lethal amounts of EV-V died, while all the mice that received EV-R survived. Severe pathogenesis correlated with viral burdens and proinflammatory cytokine levels were observed in EV-V-challenged mice, but controversy in EV-R-challenged mice. Consensus sequence analysis revealed EV-R rapidly acquired complete mutations at E145G and S241L and partial mutations at V146I of VP1, and acquired a T to C substitution at nucleotide 494 of the 5′-UTR. EV-R exhibited higher binding affinity for another EV71 receptor, human P-selectin glycoprotein ligand-1 (hPSGL-1), than EV-V. Both EV71s exhibited no significant difference in binding to hSCARB2. The molecular modelling indicate that these mutations might influence EV71 engagement with PSGL-1 and *in vivo* virulence.

## Introduction

Enterovirus 71 (EV71) has emerged as a serious threat to public health, causing hand, foot and mouth disease (HFMD) and herpangina across the Asia-Pacific region^1–3^. Severe neurological disorders, including encephalitis, acute flaccid paralysis, pulmonary oedema (PE), and haemorrhaging, culminating in death, particularly in EV71-infected children under 5 years old, have been reported^1–5^.

Enterovirus is a single-stranded RNA virus of the *Picornaviridae* family. Its single open reading frame codes for a polyprotein that contains three regions, P1, P2 and P3. When it infects cells, the P1 precursor, which is encoded by the P1 region, is cleaved by the protease into VP1, VP2, VP3 and VP4; then, VP1, VP2, and VP3 are exposed on the surface of virions that are responsible for host-receptor binding^6^. Along with other attachment receptors, including annexin-2, sialylated glycan, heparin sulfate, and heat shock protein 90^7–10^, two human functional receptors, human P-selectin glycoprotein ligand-1 (PSGL-1) and human scavenger receptor class B 2 (hSCARB2)^6,11^, have been identified to be used in EV71 and CVA16 infections; then, infected cells produce non-infectious empty (E)-particles or infectious full (F)-particles^12,13^. Transgenic mice expressing hSCARB2 develop HFMD-like skin rashes and severe limb paralysis and death after inoculation with EV71 or CVA16^14^.

The passage of isolated virus in animals and cell cultures may induce mutations that alter viral virulence or tropism^15^. Among the capsid proteins of EV71, VP1 plays a central role in particle assembly and cell entry^16^, and is used in viral identification and evolutionary analysis. Mutations in VP1 affect virus-receptor binding ability and virulence^17–20^ and even allow the virus to escape the host immune response^21,22^. The L97R amino acid mutation in VP1 enhances the neuronal tropism of EV71^23^. Changing the accessibility of the positively charged lysine side chain of the VP1 244 residue may affect the interaction between the VP1 145 residue and the negatively charged N-terminus of PSGL-1 to modulate virus tropism in leukocytes^17^. Examination of capsid sequences of representative EV71 strains revealed that PSGL-1-binding viruses have either a G or a Q at residue 145 within the VP1 capsid protein (VP1-145G or Q), whereas PSGL-1-nonbinding viruses have VP1–145E^17^, which has been implicated as a determinant of virulence in humans^24,25^. Moreover, BPR0Z-194, one of the pyridyl imidazolidinones, is a selective EV71 inhibitor that targets VP1, and the V192M single mutation in VP1 confers resistance to these inhibitory effects^26^. The suramin analogue NF449 inhibits EV71 binding to cell surface, and resistance to NF449 may occur in viruses that have both the E98Q and K244R mutations in VP1^27^. Mutations at residues I113 and V123 in VP1generate resistance to two novel capsid-binding compounds, the pyridyl imidazolidinones analogues NLD and ALD, during serial passage of EV71^28^.

The 5′-untranslated region (5′-UTR) of enterovirus RNA is fundamental for initiating translation, as it interacts with cellular RNA binding proteins to produce the viral polyprotein during replication^29,30^; the initiation of translation at the 5′-UTR involves internal ribosome entry sites (IRESs) that occupy most of the rest of the viral 5′-UTR^31^. The 5′-UTR of EV71 participates in virus replication by interacting with poly(C)-binding protein 1 (PCBP1)^32^, and a single nucleotide change from cytosine to uracil at base 158 of the 5′-UTR contributes to reduced EV71viral translation and virulence in mice^33^. Heterogeneous nuclear ribonucleoproteinA1 was reported to bind to the 5′-UTR of EV71 and regulate IRES-dependent translation^34^.

Here, we report that the presence of the mutations E145G, V146I, and S241L in the VP1 sequence and a single nucleotide T to C mutation at nucleotide 494 in the 5′-UTR alter virulence during EV71 propagation. Parental EV-V, which was propagated in Vero cells, exhibits virulence in cell culture and severe limb paralysis resulting in death in hSCARB2-Tg mice^14^; in comparison, EV-R, which was generated from the passage of EV-V in RD cells for very short generations, exhibits reduced virulence and pathology in mice. This evidence indicates that EV-R exhibits greater binding to PSGL-1 and has lost its ability to infect and cause pathology in animals.

## Materials and Methods

### Ethics statement

All animal experiments followed the guidelines of the Laboratory Animal Center of the National Health Research Institutes (NHRI), Taiwan (http://lac.nhri.org.tw/e_index.php). The animal protocols were reviewed and approved by the NHRI Institutional Animal Care and Use Committee (Approval Protocol No. NHRI-IACUC-104120-A). At the end of the study, the tested animals were euthanized by 100% CO_2_ inhalation for 5 min followed by cervical dislocation to minimize suffering.

### Cells, viruses, drugs, and antibodies

African green monkey kidney (Vero; ATCC No. CCL-81) cells were provided by the Taiwan Centers of Disease Control; the original cell lines were obtained from the American Type Culture Collection (ATCC, United States). NIH3T3 mouse fibroblast cells (ATCC No. CRL-1658) were purchased from ATCC. Human rhabdomyosarcoma (RD; BCRC No.60113) cells were purchased from the Bioresource Collection and Research Center (BCRC) of Taiwan. 3T3-SCARB2 cells, which are derived from NIH3T3 transfected with a plasmid carrying human SCARB2 cDNA to express hSCARB2 (3T3-SCARB2), were generated in our previous work^35^. Mouse L929 cells transfected with the pEF-PSGL-1, a pEF6-Flag-3S vector carrying the cDNA encoding human PSGL-1, to obtain a human PSGL-1 expressing stable clone (PSGL-1-L929) and L929 (Bsd) mouse cells transfected with pEF-bsd, an empty vector of pEF6-Flag-3S, were generously provided by Dr. Yorihiro Nishimura of the Department of Virology II at the National Institute of Infectious Diseases, Musashimurayama-shi, Tokyo, Japan^6^.

Vero cells were cultured in VP-SFM medium (Gibco-Invitrogen, CA, USA) supplemented with 4 mM L-glutamine (Gibco-Invitrogen, CA, USA). 293A (Invitrogen- Thermo Fisher Scientific, CA, USA), RD, and NIH3T3 cells were cultured in DMEM medium containing 10% foetal bovine serum (FBS, Gibco-Invitrogen, CA, USA). NIH3T3-SCARB2 cells were maintained in DMEM medium supplemented with 10% FBS and 800 mg/mL of G418 (Sigma-Aldrich, Co. LLC, USA). L929 (Bsd) and L929-PSGL-1 cells were maintained in the previously described DMEM medium, to which 5 µg/ml blasticidin S-HCl (Sigma-Aldrich, Co. LLC, USA) was added. Cells were maintained in a 37 °C incubator equilibrated with 5% CO_2_. A clinically isolated strain of EV71, Tainan/5746/98(C2)(GenBank: AF304457.1), was obtained from Dr. Jen-Ren Wang of National Cheng-Kung University, Tainan, Taiwan and was propagated in Vero cells (called as EV-V)^36,37^. EV-V was further passaged 2 to 3 times in RD or 293A cells to produce EV-R or EV-293A, respectively (Supplementary Table S3). The EV71 generated was concentrated using the hollow fibre method (Spectrum, D02-E100-05-N) with the MAP-TFF system (Lefo Science, MAP01-TFF). Virus stocks were stored at −80 °C, viral titres were determined in RD cell using a standard plaque-forming assay^35^, and the number of plaque-forming units (pfu) was calculated.

The Mab979 monoclonal antibody, which recognizes the VP0/VP2 capsid protein of EV71^38^, was purchased from Millipore, Inc. (MA, USA). A VP1-specific monoclonal antibody E1 was produced in-house, as described previously^38^. Horseradish peroxidase (HRP)-conjugated donkey anti-mouse antibody (Cat. No. 715-036-150) and HRP-conjugated rabbit anti-goat antibody (Cat. No.305-035-003) were purchased from Jackson ImmunoResearch, Inc. (PA, USA).

### Challenge of hSCARB2-transgenic mice with EV71 mutants

hSCARB2-Tg mice on a C57BL/6 background were previously generated by our group and were maintained by cross-mating hSCARB2-Tg individuals to obtain inbred mice^14^. Fourteen-day-old hSCARB2-Tg mice were inoculated s.c. with PBS, 5 × 10^6^ pfu of EV-V or 5 × 10^6^ pfu of EV-R, and the mice were monitored daily for survival for 15 days after challenge. Scoring of neurological diseases in the mice was followed by our previous study^14^; 5 = severe front and rear limb paralysis (LP) and no movement, 4 = moderate 2 rear LP and hesitant movement, 3 = one rear LP with bending legs, 2 = mild rear limb bended, 1 = slightly rear limb bended, 0 = normal movement. LP is defined as the rigidness of mouse legs which are hesitate to move.

### Immunohistochemical staining

The spine and surrounding muscle tissues of hSCARB2-Tg mice infected with or without 5 × 10^6^ pfu of EV-V or EV-R were isolated on day 4 post infection (pi), placed directly into 10% formaldehyde (Sigma-Aldrich) solution overnight and embedded in paraffin for sectioning. Four-micron sections were slided (Leica CM1800) and placed on poly-L-lysine-coated glass slides before fixation with 3–7% paraformaldehyde. The slides were stained with H&E or with the anti-EV71 antibody (Mab979, Millipore). Sections from uninfected Tg mice incubated with a primary antibody were included as a negative control. The slides were washed with PBS and then incubated with biotinylated antibody followed by staining with the Ultra-Sensitive ABC Mouse IgG staining Kit (Thermo Scientific). A red-to-brown peroxidase stain was developed using the DAB PLUS substrate kit (Zymed Laboratories). A digital slide scanner (3DHISTECH, Pannoramic MIDI) was used to scan the entire slide and then transform the image into digital files. Images captured at 10× magnification were analysed with Pannoramic Viewer software (3DHISTECH).

### Real time RT-PCR

Total RNA was purified from tissues using RNAzol reagent (Invitrogen, CA, USA) according to the manufacturer’s instructions and then subjected to real-time reverse transcription-polymerase chain reaction (RT-PCR). Total RNA was converted into cDNA using random primers (Genomics BioSci&Tech, Taiwan) plus reverse transcriptase (HiScript I, Bionovas, Toronto, Canada). The resulting cDNAs were then subjected to quantitative real-time PCR analysis (LightCycler 480 SYBR Green Real-Time PCR system) with EV71 VP1 or proinflammatory cytokine-specific primers were measured. Mouse β-actin genes in the samples were used as the internal control. The reaction in the well without RNA was treated as the background control. The PCR conditions were as follows: 95 °C for 3 min; 40 cycles at 95 °C for 10 s, 65 °C for 20 s, and 72 °C for 2 s; and a final incubation at 72 °C for 2 min. The forward and reverse primer sets used to amplify and detect VP1, mouse β-actin, and CXCL10, CCL3, and TNF-α, which were the same as those previously noted in^14^, are listed in Supplementary Table S1. To determine the relative expression of VP1, the individual Ct obtained from EV-V- or EV-R-infected tissues was normalized by subtracting its respective Ct (β-actin), and then each value of 2^Normalized mean Ct (the tissue of Tg mouse infected with EV-V or Mock)^ was divided by the value of the mean of 2^Normalized Ct (the tissue of Tg mouse infected with EV-R)^. To determine the relative expression of the chemokines, each value of 2^Normalized mean Ct (target gene from the tissue of Tg mouse infected with EV71)^ was divided by the value of the mean of 2^Normalized Ct (respective tissue of EV71-infected Tg without RNA)^. All primer sets were commercially synthesized by Genomics BioSci&Tech, Taiwan.

### EV71 nucleotide sequence analyses

Genomic RNA was extracted from 5 × 10^6^ pfu of EV71 using RNAzol reagent (Molecular Research). Reverse transcription was subsequently performed using random primers plus reverse transcriptase to obtain viral cDNA. The resulting cDNA was amplified by PCR with Phusion polymerase (Thermo) with serial primers targeting the entire genome of EV-V and EV-R. Alternatively, the nucleotide sequence of VP1 region in EV-293A was also analyzed (Supplementary Table S2). The sequencing reactions were performed by Genomics BioSci&Tech, Taiwan.

### Cryo electron microscopy (cryoEM)

Purified EV-V and EV-R were inactivated by UV light exposure and prepared using a standard cryoEM procedure. In short, one drop (~3 µL) of inactive virus in 2% glutaraldehyde buffer was applied to glow-discharged TEM copper carbon-coated grids (Electron Microscopy Sciences) and mounted onto the plunge freezing device (Gatan CP3). The sample with the grid was frozen in the liquid ethane and then transferred to liquid nitrogen for further image collection. TEM imaging was performed with a JEOL1400 TEM at a magnification of 30,000× and an acceleration voltage of 120 kV. All digital images were acquired with a Gatan, Inc. Ultrascan 4000 4 k × 4 k CCD Camera System (Model 895). Virions harvested from RD and Vero cells were selected manually using the *e2boxer.py* package in EMAN2. ML2D, an image classification algorithm package included in Xmipp 3.1, was used to classify the selected particles as empty or full. The crystal structure of the EV-VP1 protein (PDB ID code: 3VBS) was manually fitted into the mature EV71 cryoEM structure (EMDB ID code: 5558)^39^ using UCSF Chimera.

### Enzyme-linked immunosorbent assays (ELISAs)

To detect the binding activity of EV71 variants to hSCARB2 and hPSGL-1, 96-well plates (Corning, Cat. No. 9018) were coated with100 μL per well of various amounts of recombinant proteins (Sino Biological Inc.) or bovine serum albumin (BSA; Sigma, Cat. No. A9647) in 0.1 M Na_2_HPO_4_ (J.T.Baker, Cat. No. 3828) and incubated at 4 °C overnight. Plates were washed three times with PBS and then blocked with 4% skim milk/PBS buffer at room temperature for one hour. One hundred microliters of 5 × 10^4^ or 1 × 10^5^ pfu of EV71was added to each well of plates containing immobilized PSGL-1 or SCARB2, respectively, and the plates were incubated at 37 °C for 2 hours. Plates were placed in the equipment (Stratalinker®, UV Crosslinker Model 2400) and exposed twice for UV cross-linking. The plates were washed with PBS, 100 µl of 1:1000 diluted mouse anti-EV71 IgG antibody (Mab979; Millipore) was added to each well, and the plates were incubated at room template for one hour. After three washes with PBS, 100 µlof diluted biotinylated goat anti-mouse antibody (1:1000; Thermo Fisher, Cat. No. 1852330) was added to each well for one hour. One hundred microliters of 1:1000 diluted HRP-streptavidin conjugate (Invitrogen, Cat. No. 43-4323) was added to each well, and the plates were incubated for another hour after being washed three times with PBS. The reaction was developed by incubation with 100 μL of 3,3′,5,5′-tetramethylbenzidine (TMB; KPL, Cat. No. 52-00-03) substrate for 20 min in the dark and terminated by adding 50 μL 2 N H_2_SO_4_. The optical density at 450 nm was determined using a microplate absorbance reader (SPECTRA, MAX2, M2). The limit of detection for this assay is 0.03 at OD_450_.

### Viral entry and replication assays

L929-PSGL-1 or 3T3-SCARB2 cells (4 × 10^5^ per well) were cultured in 6-well plates at 37 °C overnight and subsequently infected separately with EV-R or EV-Vat an MOI = 0.1. Cells were washed twice with DMEM after 1 hour of infection and were cultured in 2 mL DMEM with 10% FBS at 37 °C. For viral entry assay, cells were harvested, and total RNA was extracted by adding RNAzol at 1 and 3 hours post infection (pi). For the viral replication assay, cells were collected for the plaque assay at 12, 24, and 48 hours pi.

### Statistical analysis

**Figure 1:**
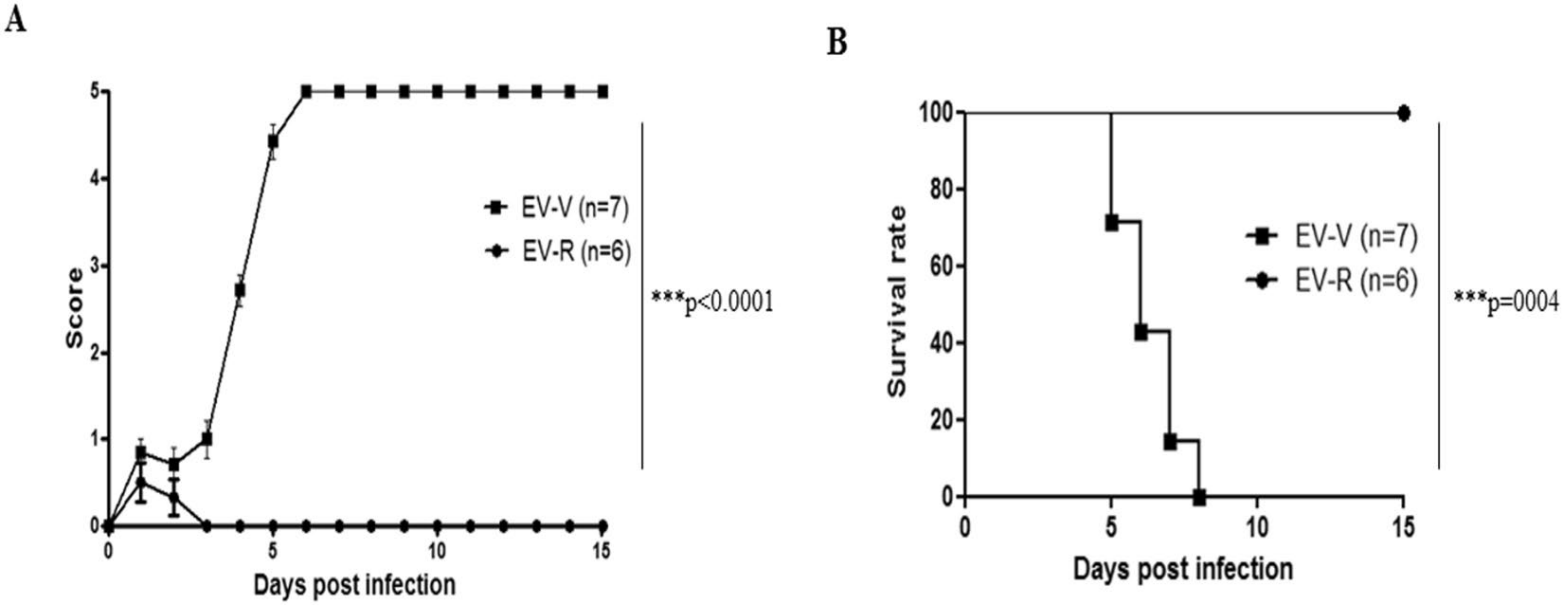
Challenging hSCARB2-Tg mice with EV-V, but not EV-R, causes lethal pathogenesis. Fourteen-day-old hSCARB2-Tg mice were challenged s.c. with 5 × 10^6^ pfu of EV-V (■) or EV-R (●), and animals were monitored daily for (**A**) scoring of CNS-related paralysis and (**B**) survival for 15 days. The number (N) of transgenic mice per group is shown. Two-way ANOVA and log-rank test were used for statistical analysis in (**A** and **B**), respectively.

A software of GraphPad Prism for statistical analysis was used in whole manuscript. Two-way ANOVA and log-rank test were used in Fig. 1A,B, respectively. One tail unpaired student’s t-test was used in Figs 3–5. The results were considered statistically significant when *P* < 0.05. The symbols *, **, and *** are used to indicate *P* < 0.05, *P* < 0.01, and *P* < 0.001, respectively.

## Results

### EV71 virulence in hSCARB2-Tg mice differs after propagation in RD cells *vs*. Vero cells

**Figure 2:**
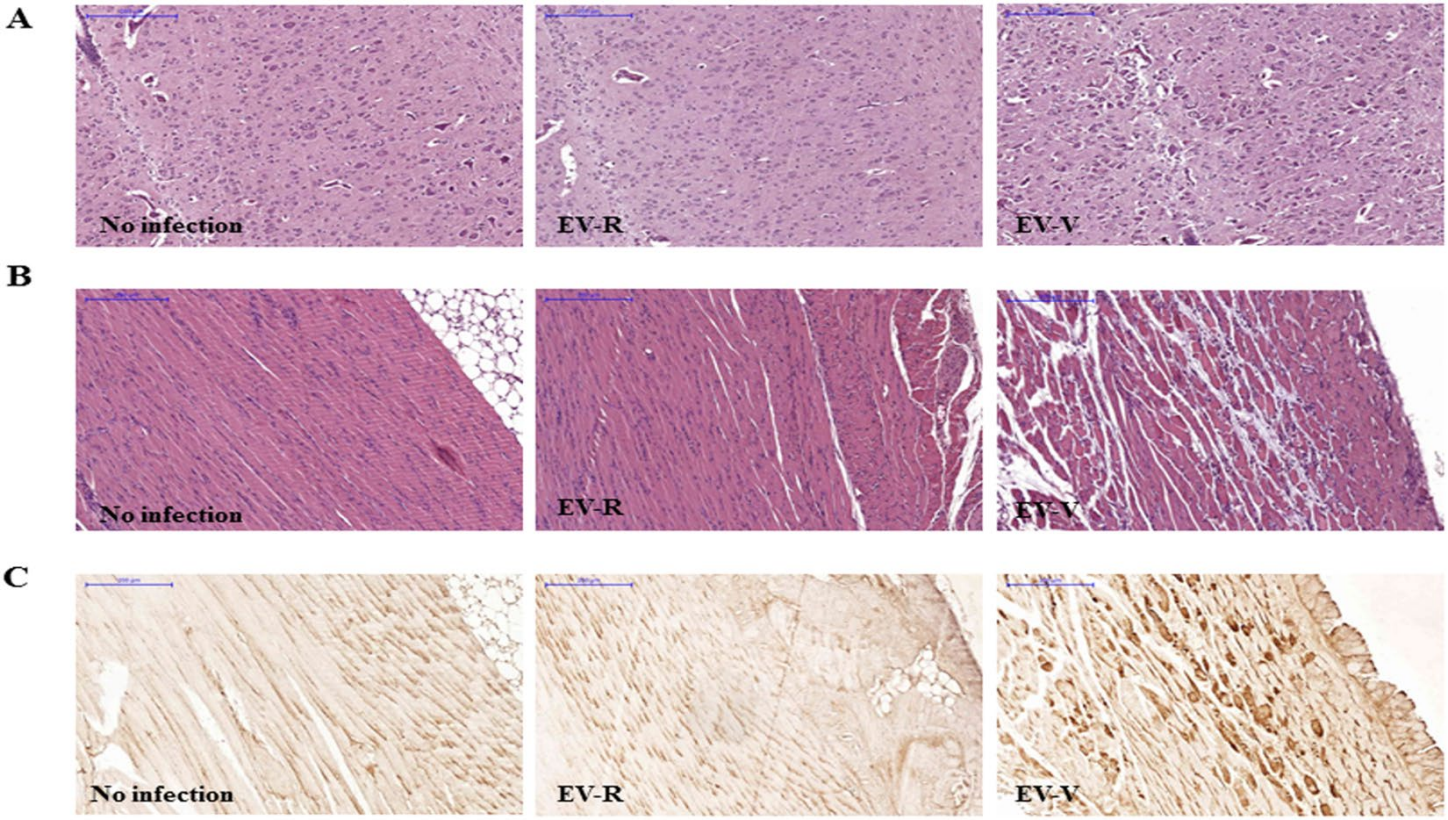
Tissue pathogenesis is induced by EV-V, but not EV-R, in hSCARB2-Tg mice.Serial sections of spinal cord and the surrounding muscle from naïve mice and hSCARB2-Tg mice challenged with 5 × 10^6^ pfu of EV-V or EV-R were obtained on day 4 pi and then stained with dyes or antibodies. Representative images of H/E-stained (**A**) spinal and (**B**) muscle sections are shown. (**C**) Representative images of VP2 of EV71 in respective muscle sections that were stained with Mab979, a VP2-specific antibody. All images were obtained at 10× magnification, and the scale bars represent 200 µm. Similar results were obtained from two independent experiments with four mice per group; one is shown.

**Figure 3:**
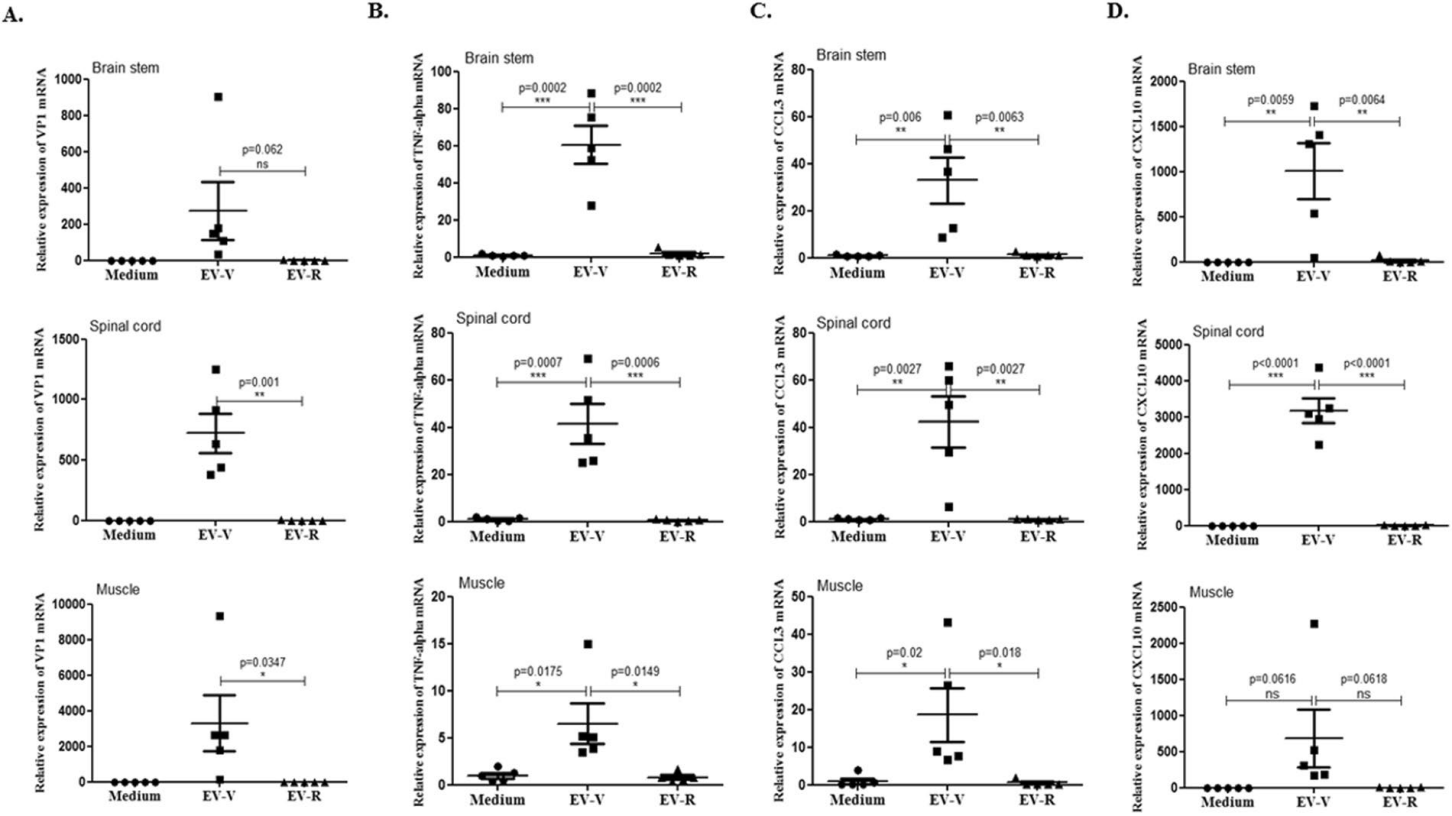
Expression of VP1 and proinflammatory cytokines in the tissues of EV71-infected Tg-mice. Four days afterchallenginghSCARB2-Tg mice with 5 × 10^6^ pfu of EV-V or EV-R s.c., RNA was extracted from the brainstem, spinal cord, and muscle, and quantitative RT-PCR analysis was conducted to quantify (**A**) VP1, (**B**) TNF-α, (**C**)CCL3, and (**D**) CXCL10 expression. hSCARB2-Tg mice that received medium were used as the negative control. The number of PCR cycles required for fluorescent detection of target genes was calculated and is presented as the relative expression after normalization to the internal control of β-actin expression from the respective tissue as described in the Materials and Methods. A schematic representation of target gene expression and the statistical average from 5 mice per group are shown. One tail unpaired student’s t-test was used for statistical analysis.

The propagation of the clinically isolated EV71 in Vero or RD cells has been reported previously^11,40^. To evaluate the pathogenesis of EV71 *in vivo*, the C2 genotype of EV71 Tainan/5746/98 was cultured in Vero cells (EV-V), or viral seeds were passaged in RD cells to generate EV-R pathogens (Supplementary Table S3). The yield of EV-V was steadily produced after three passages (P); 5.2 × 10^5^, 8.9 × 10^5^, and 7.8 × 10^5^ pfu/mL of P1, P2, and P3 viruses, respectively, was detected. In contrast, EV-R was adapted in RD cells and turned to higher (10-fold) replication at P2 and then steady at P3; 4.0 × 10^5^, 4.3 × 10^6^, and 6.9 × 10^6^ pfu/mL of P1, P2, and P3 viruses, respectively, were detected. The pictures indicated the cytopathic effect of EV-V *vs*. EV-R in each passage were no significant difference (Supplementary Table S3). hSCARB2-Tg mice on a C57BL/6 background were shown to be an experimental model that develops HFMD-like disease after infection with the B2 and C2 genotypes of EV71 (those propagated in Vero), with severe CNS pathology leading to death^14^. Our previous study had shown that infection of EV-V induced a mild or no pathogenic responses in 2-week-old C57BL/6 (non-Tg) or in adult hSCARB2-Tg mice, respectively, compared to severe disease in 2-week-old hSCARB2-Tg^14^. We examined the pathogenesis of EV-V and EV-R in this model and confirmed that EV-V caused severe paralysis (Fig. 1A) that resulted in death at 3 × 10^6^ pfu (Fig. 1B). However, the same amounts of EV-R infection only caused mild symptoms, and animals recovered by day 10 after infection (Fig. 1A,B). We therefore examined the pathogenesis in spinal cords and the surrounding muscles that were sectioned from normal, EV-R, and EV-V-infected Tg mice on day 4 pi (Supplementary Fig. S1). As shown in Fig. 2A,B, H/E histochemical staining of spinal cords and the surrounding muscle revealed that the cellularity of the spinal cord was slightly increased in EV-V-infected animals, and multiple foci of myositis with moderate lymphocyte infiltration and muscle cell destruction were found near the spine. However, only mild myopathy was observed in EV-R-infected animals. Sections taken from corresponding sites in naive mice appeared normal. The respective serial-sections were also immunostained with an anti-VP2 antibody, with a strong viral antigen signal being observed in EV-V-infected muscle compared to the background signals detected in naive and EV-R-challenged tissues (Fig. 2C). This result corresponded to the tissue viral loads measured with real-time RT-PCR of VP1; more EV-V than EV-R accumulated in the brainstem, spinal cord, and muscle of infected Tg mice (Fig. 3A).

The excessive release of IL-6, TNF-α, IL-1β, CXCL10, and IFN-γ in EV71 patients experiencing pulmonary oedema^2,41–43^ and upregulation of CXCL10 and CCL3 in the CNS and peripheral tissues of EV71-infected hSCARB2-Tg mice^14^ have been reported. We therefore measured the expression of proinflammatory mediators in the tissues of Tg mice after EV-V or EV-R infection. Real-time RT-PCR analysis confirmed that EV-V, but not EV-R, significantly induced the expression of TNF-α, CCL3, and CXCL10 in the brainstem, spinal cord, and muscle (Fig. 3B–D, respectively). These results indicate that the severity of pathogenesis induced by virulent EV-Vis correlated with the accumulated viral loads and proinflammatory cytokine levels in these tissues.

### VP1 amino acid substitutions and a single mutation in the 5′-UTR are present in Vero *vs*. RD-propagated EV71

Previous results have indicated that the EV71 capsid region determines viral infectivity^18^. To study whether the differences in the virulence of EV-V and EV-R are associated with amino acid mutations, we sequenced total viral cDNA from EV-V and EV-R and compared these sequences to those reported in the database for Tainan/5746/98 (GenBank: AF304457.1). Alignment of the individual nucleotides of EV-V and EV-R revealed alterations in the encoded amino acids at residues 104, 145, 146, and 241 of VP1 (Supplementary Fig. S2A–D, respectively). The results summarized in Table 1 show that residesE145G and S241L were completely mutated in EV-R relative to EV-V, but residue V146I was partially mutated in EV-R. VP1-145 and neighbouring residue 146 are reportedly located in between a β-sheet D site (βD; amino acids 130–141) and a βE site (amino acids 149–156), and VP1–241 is located after a βH site (amino acids 232–236) in VP1^44^. In addition to these mutations, the VP1–104 residue was partially substituted in EV-V; Asp and Asn were identified in EV-V, but only Asn was found in EV-R. The T nucleotide at the 5′-UTR 494 site in EV-V was found to be converted to a C nucleotide in EV-R (Table 1 and Supplementary Fig. S2E). To investigate this phenomenon occured in general or just happened by chance, EV-V were cultured in 293A cells to produce EV-293A and it’s cDNA was sequenced and compared these sequences to EV-V. One residue were mutated from E to Q in VP1–145 of EV-293A relative to EV-V (Table 1). These results indicate that some specific nucleotides in VP1 and the 5′-UTR of EV71 can quickly mutate from the resides found in Vero cells during the last 2–3 runs of passage RD or 293A cells to adapt to the host environment; however, these changes may come at the expense of virulence.

**Table 1:** Analysis of mutations in EV-V *vs*. EV-R *vs*. EV-293A.

Mutation Sites	VP1 104	VP1 145	VP1 146	VP1 241	5′-UTR 494
*Database	AAT Asn	GAA Glu	GTT Val	TCG Ser	T
EV-V	AAT Asn GAT Asp	GAA Glu	GTT Val	TCG Ser	T
EV-R	AAT Asn	GGA Gly	GTT Val ATT Ile	TTG Leu	C
EV-293A	AAT Asn	CAA Gln	GTT Val	TCG Ser	ND

^*^Site specific nucleotide and the encoded amino acid sequence of EV71 Tainan/5746/98 (GenBank: AF304457.1) as reference was shown. ND: detection was not performed.

### Alterations in receptor binding determine EV71 entry and replication in susceptible cells

**Figure 4:**
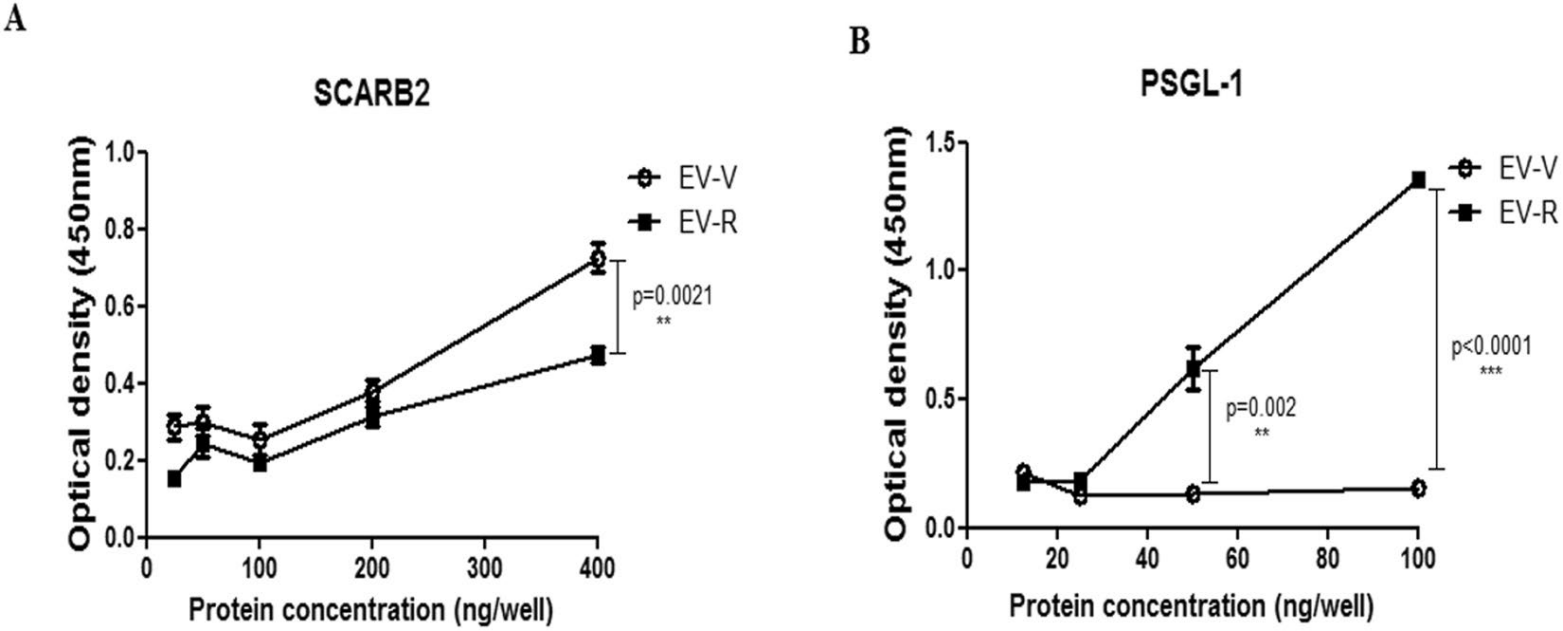
Binding activity of EV-R *vs*. EV-V in the interaction of human PSGL-1 and SCARB2. Ninety-six-well plates coated with live EV-V or EV-R (10^3^ pfu) were incubated with various amounts of recombinant (**A**) hSCARB2-Fc or (**B**) hPSGL-1-Fc per well for 1 hour at room temperature. After incubation, the plates were washed three times with PBS, and then the EV71 particles in the wells were quantified by incubating with MAB979 antibody in an ELISA assay as described in the Materials and Methods. Three independent experiments were performed, and one of results is shown. One tail unpaired student’s t-test was used for statistical analysis.

**Figure 5:**
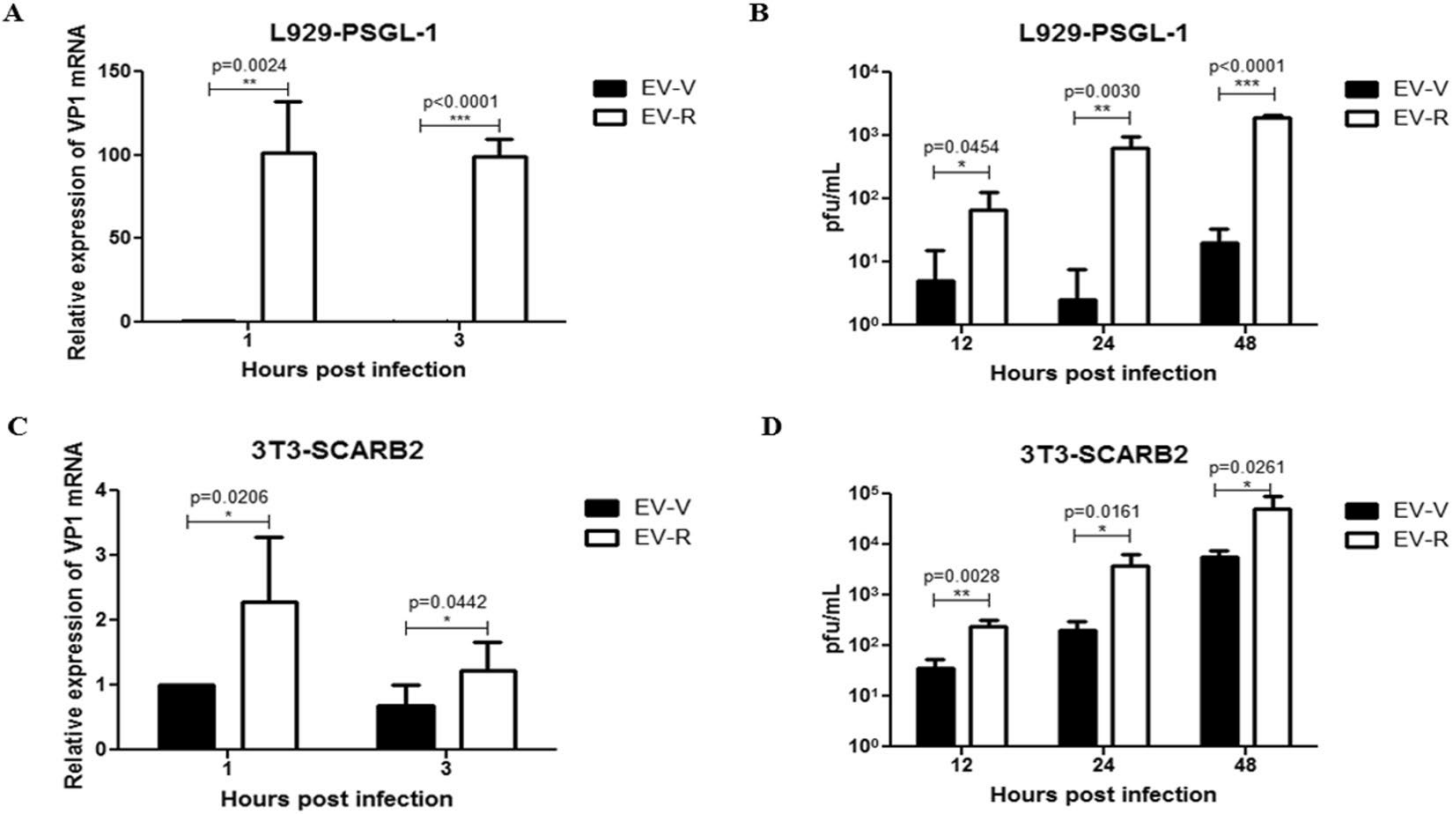
Susceptibility of EV-R *vs*. EV-V in human receptor-expressing mouse cells. L929-PSGL-1 and NIH3T3-SCARB2 cells (4 × 10^5^ per well) were cultured in 6-well plates overnight and subsequently infected separately with EV-R or EV-Vat an MOI = 0.1. Cells were washed after 1 hour of infection and cultured for another 1 or 3 hrs for viral RNA extraction or 12, 24, or 48 hrs for a plaque-forming assay. (**A**,**C**) RNA was extracted from EV71-infected (**A**) L929-PSGL-1 or (**C**) 3T3-SCARB2 cells and subjected to quantification of EV71 transcripts with real-time RT-PCR using specific primers against the VP1 region and β-actin. The relative expression of the target gene was calculated as described in the Materials and Methods. Each VP1 2^Ct^ value was normalized by calculating the ratio to the respective β-actin 2^Ct^ value, and then the means of normalized 2^Ct^ values were calculated. A schematic representation of the expression ofEV71 transcripts is shown. (**B,D**) The supernatants from EV71-infected (**B**) L929-PSGL-1 or (**D**) 3T3-SCARB2 cells were harvested and used to assay for viral titre with a plaque-forming assay as described in the Materials and Methods. Data represent one of three independent experiments. One tail unpaired student’s t-test was used for statistical analysis.

EV71 has been reported to bind to SCARB2 via a cleft in VP1 around residue Gln-172, but not Q145^45^. In addition, the VP1–145 residue is a critical molecular determinant for EV71 binding to PSGL-1^17^. We speculated that mutations in this residue in VP1 might change the structural conformation of the capsid and thus alter the ability of viruses to bind to the cellular receptor. We therefore examined the interaction between EV71 mutants and human PSGL-1 or SCARB2 by evaluating the binding of viruses with varying amounts of recombinant human PSGL-1 or SCARB2 fused to the Fc region of human IgG1 (PSGL-1-Fc or SCARB2-Fc, respectively, as described previously)^46^ using an ELISA binding assay. Our results indicated that EV-V exhibits more interactions with SCARB2 than EV-R, even when highest amount of SCARB2was added in the assay (Fig. 4A). In contrast, EV-R exhibited a significantly higher affinity than EV-V in binding to PSGL-1 (Fig. 4B). We further assayed the infectivity of EV71 mutants in specific receptor-expressing L929-PSGL-1 cells, which are mouse fibroblastL929 cells expressing human PSGL-1^47^, and 3T3-SCARB2 cells, which are mouse NIH3T3 cells expressing human SCARB2^35^. Total RNA was harvested from the cells infected with EV71 variants at 1 and 3 hours pi and then subjected to RT-PCR reaction using primers targeting VP1. Subsequently, viral production was examined with a plaque-forming assay at 12, 24, and 36 hours pi. The amount of viral RNA (Fig. 5A) and number of forming particles (Fig. 5B) detected were drastically greater in EV-R-infected L929-PSGL-1 cells (>100-fold) than EV-V-infected L929-PSGL-1 cells. These observations were also made in 3T3-SCARB2 cells, but the increase in levels of viral RNA and the number of EV-R particles produced relative to EV-V-infected cells was not as great (<10-fold, Fig. 5C,D). Parental L929, Bsd-1, and NIH3T3 cells were all observed to be resistant to both EV71 infections (data not shown). These results suggest a correlation between the greater EV-R tropism observed in PSGL-1-expressing cells and higher binding activity with PSGL-1, but less of a difference between EV-V and EV-R interactions with SCARB2 and tropism to SCARB2-expressing cells.

### Tertiary structure of EV71 virions

**Figure 6:**
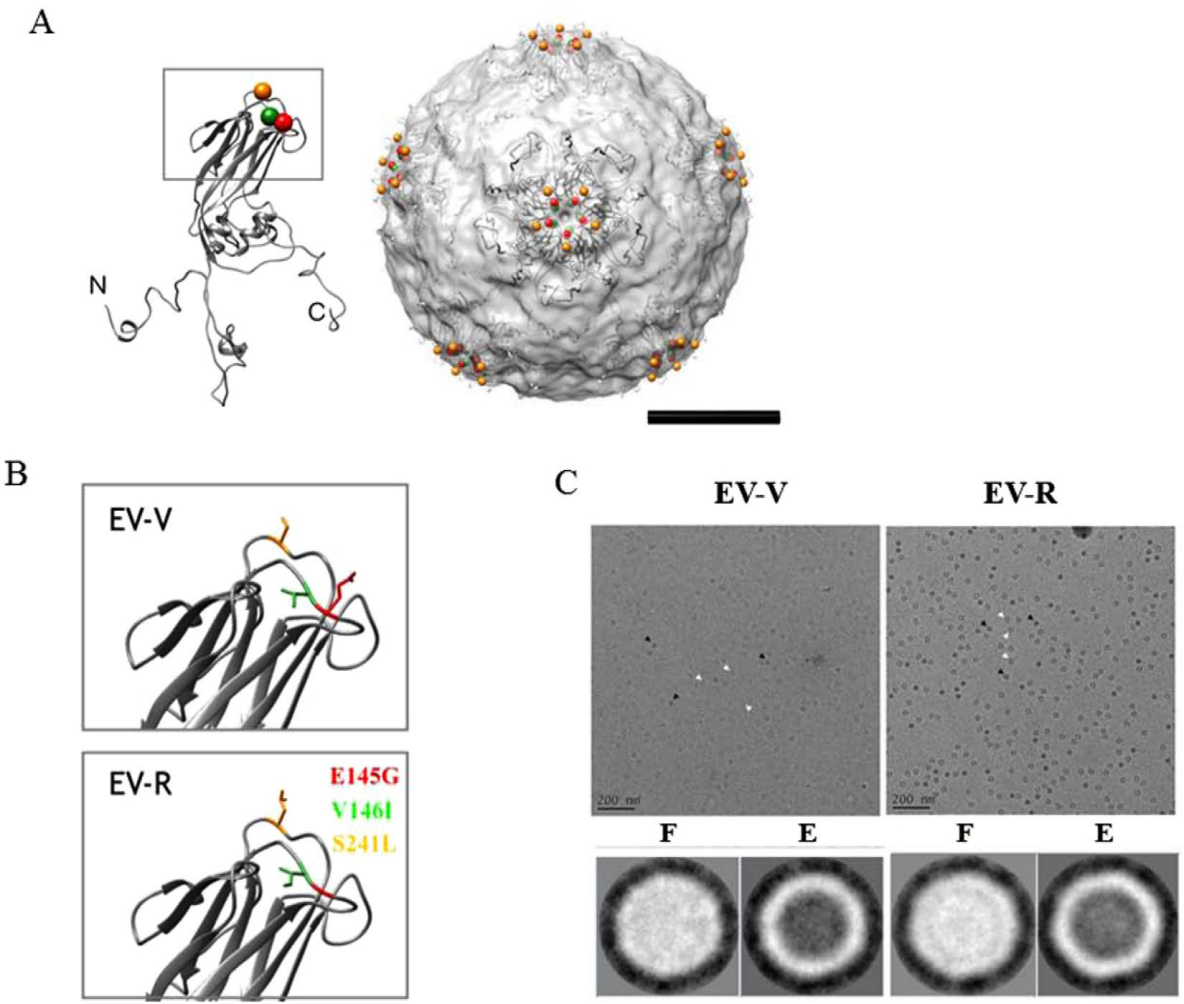
Modelling of the steric structure of EV71 particles and EV71 mutants. EV-V and EV-R particles were prepared for TEM imaging as described in the Methods. (**A**) The atomic structure of VP1 extracted from VP1-4 complex (PDB: 3VBS) was fitted into cryoEM structure of EV71 (EMDB: 5558). The spheres showed the residues 145 (colored in red), 146 (colored in green), and 241 (colored in orange) surrounding the 5-fold axis of the structure. The N, C terminus are indicated. The scale bar = 10 nm. (**B**) The 3-D configurations of the side chains of the residues at VP1-145, 146, and 241 from EV-V and EV-R are shown. The VP1 of EV-R was modelled from SWISSMODE website, a protein structure homology-modelling server. The residues were colored as previous figure. (**C**) The cryoEM images (upper panel) and 2-D classification (lower panel) of EV71 particles derived from Vero cell line (EV-V, left panel) and from RD cell lone (EV-R, right panel). The image analyses showed that the empty (**E**) and full (**F**) particles indicated by white and black arrows, respectively existed in the samples and the diameter of EV71 particles were approximately 30 nm. The scale bar in the micrography is 200 nm. The particle picking and 2-D classification analyses were done by EMAN2 and Xmipp where the contrast was inverted.

In order to visualize the amino acid substitutions position in the EV71 structure, the solved atomic structure of VP1 from VP1-VP4 complex (PDB: 3VBS) was fitted into solved cryoEM density map (EMD: 5558). In the model, the residues 145, 146 and 241 of VP1 were surrounding the 5-fold axis of the EV71 structure (Fig. 6A). Although the tertiary structure of the VP1 mainframe of EV-V and EV-R were similar according to the structure prediction, the side chain of the amino acid substitutions did not differ much in its orientation and position using SWISSMODEL (https://swissmodel.expasy.org/) (Fig. 6B), the differences in length and properties of the side chain may influence the steric hindrance between neighboring residues and lead to the changes in overall tertiary structure of the protein. A glimpse of the samples of EV-V and EV-R was done using cryoEM, the images and 2D classification analyses showed that the empty and full particles coexisted in the EV-V and EV-R sample and the EV71 particles had a diameter of ~30 nm (Fig. 6C).

## Discussion

Our present study revealed that propagation of the C2 genotype of EV71 in different host cells would force the virus to adapt to the physiological environment via VP1 amino acid mutations. We determined the virulent EV-V has three amino acid differences in VP1 compared with the attenuated variant EV-R; namely, E145G, V146I (partially), and S241L. Moreover, the VP1–104 residue encoded two different amino acids (Asn and Asp) in the wild-type EV-V, but these were mutated into the unique residue Asn in EV-R (Table 1). We sequenced another genotype, B4 of EV71 E59, which has been used as a vaccine strain^48^, and found that the VP1–145 residue encoding Gly remained the same after passage in RD and Vero cells. These phenomena have been observed in previous studies; substitution of residue 145 from E to G while passage of EV71 in RD was observed^49,50^. The dengue virus vaccine candidate parentally was grown in C6/36, primary African green monkey kidney cells, and Vero cells. It rapidly acquired a single residue (327) mutation in domain III of the envelop protein while adapting and growing in foetal rhesus lung (FRhL) cells, resulting in loss of infectivity and immunogenicity^15^. The attenuated Japanese encephalitis virus variant with increased viral fitness was obtained during cell passage in Vero cells^51^.

The residue variations in the VP1 protein may change the specificity of the VP1-receptor interaction that occurs on the surface of neural cells and therefore may play an important role in neurological complications^52^. VP1 residenes around the five-fold axis of EV71 mediate heparan sulfate interaction; lysine residues, K162, K242, and K244 in VP1 are responsible for heparin interaction^50^. *In vitro* adaption of EV71 SHA52/97 (genotype C2) in RD induced the substitutions occurred in the residues of VP1, E98K and E145G, and the heparin-binding phenotype was completely abolished afterward^50^. Also, residues 92–106 of VP1and 136–150 of VP2 are determinants for SCARB2 binding^53^, and VP1–145 is a critical residue for the binding of EV71 to PSGL-1^17^. Eighty percent of EV71 strains that have an E residue at VP-145 are non-PSGL-1-binding (non-PB) strains, and approximately 20% that have G or Q at this residue are PB strains^17^. These results correlate with our results that EV-R, which harbours VP1–145G, shows higher binding activity to PSGL-1 but shows no to very little binding activity relative to EV-V (VP1–145E; Fig. 4), and that EV-V, which is a non-PB strain, cannot infect PSGL-1-L929 cells as efficiently as EV-R, which behaves as a PB strain (Fig. 5). We demonstrated a very mild difference in SCARB2 binding between the two EV71s (Fig. 4). Because RD and Vero cells express SCARB2 only^40,45^. These mutations help EV71 to adapt and replicate in RD cells (Supplementary Table S3), but the unexpected structure change increasing of binding affinity to PSGL-1 is occurred. We will further investigate which host factors contribute to the mutations in EV-V (non-PB) in RD cells, which only express SCARB2, to produce EV-R (PB) and will investigate whether unknown receptors/factors other than SCARB2 help EV-R to infect RD cells in the next study. The mutation in 5′-UTR nucleotide 494 observed in EV-V *vs*. EV-R (Table 1) might accelerate EV-R replication in PSGL-1- or SCARB2-expressing cells (Fig. 5). Our observation in transgenic mice correlates with the results of a previous study, which found that a mutation in the 5′-UTR sequence can affect the replication and virulence of EV71 in mice^34^ and in a fatal clinical case^54^; and a similar event was also observed in CVA16 infection in neonatal mice^55^.

VP1-145E variants are mainly responsible for the development of viremia and muscle and neuron pathogenesis in a cynomolgus monkey model^56^. In our study, EV-V (VP1-145E) was more virulent than EV-R (VP1-145G) in hSCARB2-Tg mice (Fig. 1). We observed the accumulation of higher viral loads, which triggered massively proinflammatory cytokine secretions in the neurological tissue of EV-V-infected animals (Fig. 3). These results fit the clinical data indicating that the levels of cytokines involved in the cytokine storm, such as IL-6, TNF-α, and IL-1β, were best correlated with clinical severity in cases of severe EV71 encephalitis^57^. Astrocytes and microglia have been shown to secrete high levels of IL-1, TNF-α, and IL-6^58^. Recent findings indicate that PSGL-1 functions as a checkpoint in the negative regulation of T-cell homeostasis and the inflammatory response^59^. This may explain our observation that severe pathogenesis (Fig. 1) was observed in EV-V which showed a non-PB strains but not observed in the PB strain of EV-R (Fig. 4). The expression of human hPSGL-1 in mice (hPSGL-1-Tg) which developed by our and other groups^60^ is not sufficient to acquire susceptibility of EV-V or EV-R (data not shown).

Considering the protein is made of amino acids, and each amino acid has its distinct property attributable to its side chain, the differences in amino acid composition might contribute to the changes in the structure and the interactions with other molecules. The amino acid substitutions might further leading to the difference in protein function. Substitutions of VP1-145, 146 and 241 into other amino acid residues as found in this study had been described in relation to the different behaviors of the virion. In this study, we have demonstrated that the adaptation of EV71 in RD cells from parental EV71 cultured in Vero cells might result from the rapid mutation of 3 amino acid residues in VP1 and 1 nucleotide in the 5′-UTR. Variations in the VP1-145, 146 and 241 residues determine the binding of EV71 to the PSGL-1 receptor and subsequently influence viral entry into the host cell. We also speculate that a single mutation at 5′-UTR 494 might aid in adaptation and accelerate EV71 replication in order to produce more virions. We confirmed that these mutations ultimately alter EV71 virulence; EV-V is more virulent and causes paralysis and even death in infected animals. In contrast, EV-R induces very mild disease in infected Tg mice, which ultimately recovered. Examination of the infected peripheral and CNS tissues from EV-V- and EV-R-infected mice via histochemistry and quantitation of the accumulation of virions and proinflammatory cytokine levels confirmed that EV-V induces more severe pathogenesis than EV-R. Further structural comparison of EV71 derived from Vero cell and RD cell by cryoEM single particle reconstruction is needed to confirm our findings (Fig. 6). The results of this study may provide better understanding the viral behaviors in different cells which could contribute to a better cell selection in designing vaccine candidates for EV71.

## Electronic supplementary material

Supplementary information
