# Author Correction: Mutations in VP1 and 5′-UTR affect enterovirus 71 virulence

**DOI:** 10.1038/s41598-018-26833-3

**Published:** 2018-06-04

**Authors:** Ching-Kun Chang, Shang-Rung Wu, Ying-Chin Chen, Kuen-Jin Lee, Nai-Hsiang Chung, Yi-Ju Lu, Shu-Ling Yu, Chia-Chyi Liu, Yen-Hung Chow

**Affiliations:** 1 National Institute of Infectious Disease and Vaccinology, National Health Research Institutes, Zhunan, 350 Taiwan; 2 Graduate Institute of Life Science, National Defense Medical Center, Taipei, 114 Taiwan; 3 Institute of Oral Medicine, National Cheng Kung University, Tainan, 701 Taiwan; 4 Graduate Program of Biotechnology in Medicine, Institute of Molecular and Cellular Biology, National Tsing Hua University, Hsinchu, 300 Taiwan; 5 Graduate Institute of Biomedical Sciences, China Medical University, Taichung, 404 Taiwan

Correction to: *Scientific Reports* 10.1038/s41598-018-25091-7, published online 27 April 2018

In the original version of this Article, there were errors in Affiliation 2 which was incorrectly listed as ‘Graduate School of Life Science, National Defense Medical Center, Taipei, 114, Taiwan’. The correct affiliation is listed below:

‘Graduate Institute of Life Science, National Defense Medical Center, Taipei 114, Taiwan.’

These errors have now been corrected in the PDF and HTML versions of the Article, and in the accompanying Supplementary Information file.

