# Supplementary material for: Mutations in VP1 and 5′-UTR affect enterovirus 71 virulence
Source: Sci Rep. 2018 Apr 27;8:6688. doi: 10.1038/s41598-018-25091-7 (PMC5923339; doi:10.1038/s41598-018-25091-7)
Supplement: Supplementary file 1 — Supplementary information [file 41598_2018_25091_MOESM1_ESM.pdf]

Mutations in VP1 and 5'-UTR affect enterovirus 71 virulence

Ching-Kun Chang[1,2], Shang-Rung Wu[3], Ying-Chin Chen[1], Kuen-Jin Lee[3], Nai-Hsiang Chung[1,4], Yi-Ju Lu[1], Shu-Ling Yu[1,2], Chia-Chyi Liu[1], and Yen-Hung Chow[1,2,5*]

[1]National Institute of Infectious Disease and Vaccinology, National Health Research Institutes, Zhunan 350, Taiwan. [2]Graduate Institute of Life Science, National Defense Medical Center, Taipei 114, Taiwan. [3]Institute of Oral Medicine, National Cheng Kung University, Tainan 701, Taiwan.[4]Graduate Program of Biotechnology in Medicine, Institute of Molecular and Cellular Biology, National Tsing Hua University, Hsinchu 300, Taiwan. [5]Graduate Institute of Biomedical Sciences, China Medical University, Taichung 404, Taiwan.

[*]Corresponding Author, Institute of Infectious Disease and Vaccinology, National Health Research Institutes, 35 Keyan Road, Zhunan, Miaoli County 350, Taiwan.

Tel.: 886-37-246166 ext. 37738 (Y.H. Chow)

Email address: choeyenh@nhri.org.tw (Y.H. Chow)

Supplementary Table S1. List of primer sets for Q-PCR

Name	Sequence	Function
Actin_F	ACCAACTGGGACGACATGGAGAAA	Actin gene
Actin_R	TAGCACAGCCTGGATAGCAACGTA	Actin gene
VP1_F	ACGCGCAAATGCGTAGAAAGGT	VP1 gene
VP1_R	TTAGTGGCAGTTTGCCATGCGA	VP1 gene
CCL3_F	TTCTGCTGACAAGCTCACCCT	CCL3 gene
CCL3_R	ATGGCGCTGAGAAGACTTGGT	CCL3 gene
CXCL10_F	CTCTCTCCATCACTCCCCTTTAC	CXCL10 gene
CXCL10_R	ACTTAGAACTGACGAGCCTGAGC	CXCL10 gene
TNF-α_F	TCTCATGCACCACCATCAAGGACT	TNF-α gene
TNF-α_R	TTGCACCTCAGGGAAGAATCTGGA	TNF-α gene

Supplementary Table S2. List of primers used for sequencing of the EV71 genome

Name	Sequence	Name	Sequence
EV71_1+	TTAAAACAGCCTGTGGGTTGCA	EV71_4098+	AATTCAACGATGCGGCGAGTGC
EV71_692+	CACTGAAGTCTGTGATCACTCTCA	EV71_4142-	AGATCCACTCAAGCCCCTTC
EV71_779-	AACCGGAGCGTTGTGTGGATAC	EV71_4957+	GGATAGGAAGTCCAAGGTGAGA
EV71_1029-	TGCGTAGTGATGGTAGAATTGC	EV71_5002-	ACTCACTACCGTGTCCACACTG
EV71_1036+	ATGCACAGTTCCACTACCTC	EV71_5426+	AGGAACATTAGGCAGGTCCA
EV71_1589+	GGTCTGCTGGTTGTGCCTAT	EV71_5749+	CCAGTATGGGTTTTTGAACCTT
EV71_1639-	CGTCGCACCTTGGTCATAAT	EV71_5796-	ATCATAGTCCTGTGAGTGGGCT
EV71_2011+	CTTGTTGGGCCAGTTGTGC	EV71_6218+	AAGATGAGCATGGAGGATGC
EV71_2439+	AGGGAGATAGGGTGGCAGATGT	EV71_6376-	GTCCATGTAGAATTTCATCTTG
EV71_2559-	TCTAATCGATGGCTGCTTACC	EV71_6561+	ATCACTGGTTCAGCTGTT
EV71_2623+	GAGTATGATTGAGACACGGT	EV71_6605-	TTGGTAACTTGCTCCAGAACAC
EV71_3196+	CATGAGGATGAAACACGTCA	EV71_6962+	CTGGAGTTGGCAAGAACAGG
EV71_3326-	TATCCACGCCCTGACGTGTTTC	EV71_7410-	GCTATTCTGGTTATAACA
EV71_3608+	AGATACCAGTCACATCTCATGC		

Supplementary Table S3. The passage history of EV-V *vs.* EV-R

	Day 1	Day 2	Day 3
*Produced virus/ titer (pfu/mL)	Cytopathic effect in RD		
EV-R P1 / 4.0*10^5	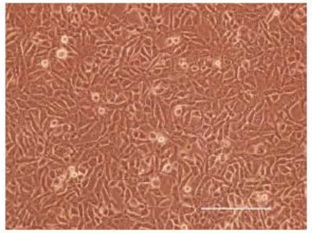	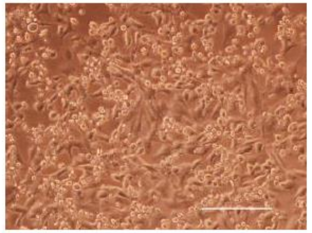	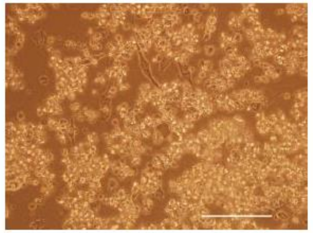
EV-R P2 / 4.3*10^6	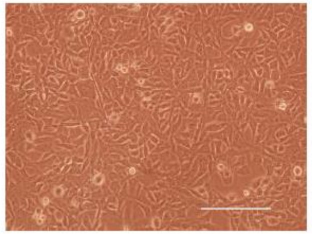	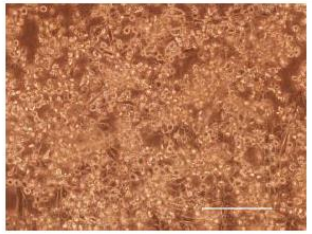	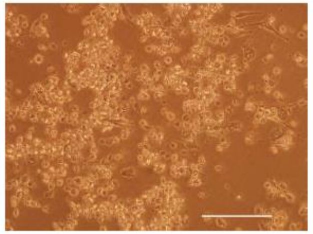
EV-R P3 / 6.9*10^6	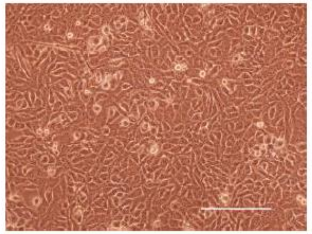	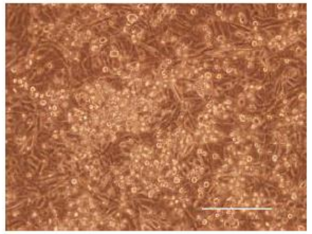	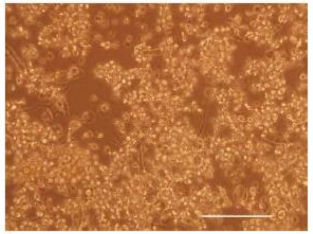

	Day 1	Day 2	Day 3
*Produced virus/ titer (pfu/mL)	Cytopathic effect in Vero		
EV-V P1 / 5.2*10^5	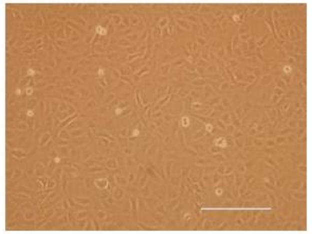	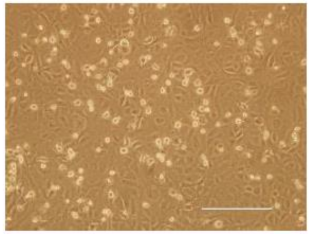	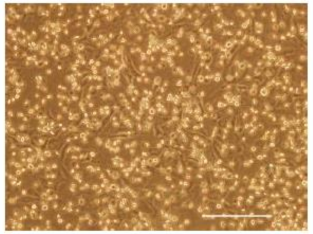
EV-V P2 / 8.9*10^5	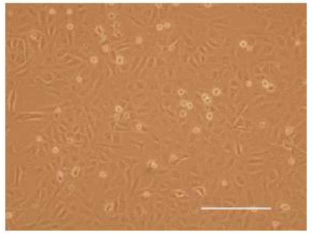	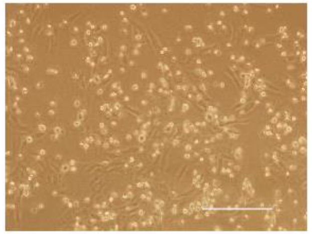	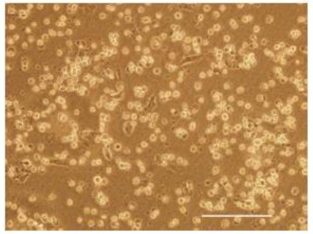
EV-V P3 / 7.8*10^5	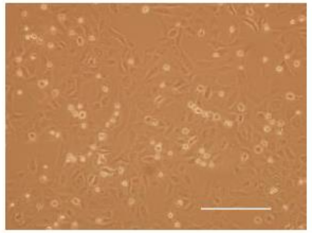	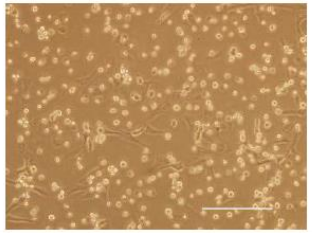	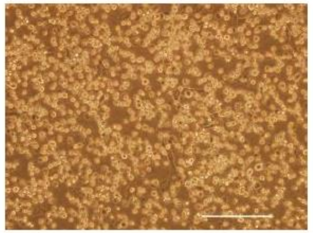

* EV71 seed (GenBank: AF304457.1) propagated from Vero cells were subjected to infect RD or Vero cells cultured in 6-well plates with the multiplicity of infection (MOI) = 0.1 (day 0) and then cultured for 3 days. The pictures showing the cytopathic effect were taken by the microscope at 20x magnitude every day. The scale bar = 200 µm were indicated. Produced EV-R P1 or EV-V P1 were collected from the supernatant of infected RD or Vero cells, respectively, at day 3 and then assayed their titer by plaque assay which performed in RD cells. EV-R P1 or EV-V P1 were used to infect RD or Vero cells to produce EV-R P2 or EV-V P2, respectively, as followed as the procedure described above. The third run of infection to produce EV-R P3 or EV-V P3, respectively, was performed as well.

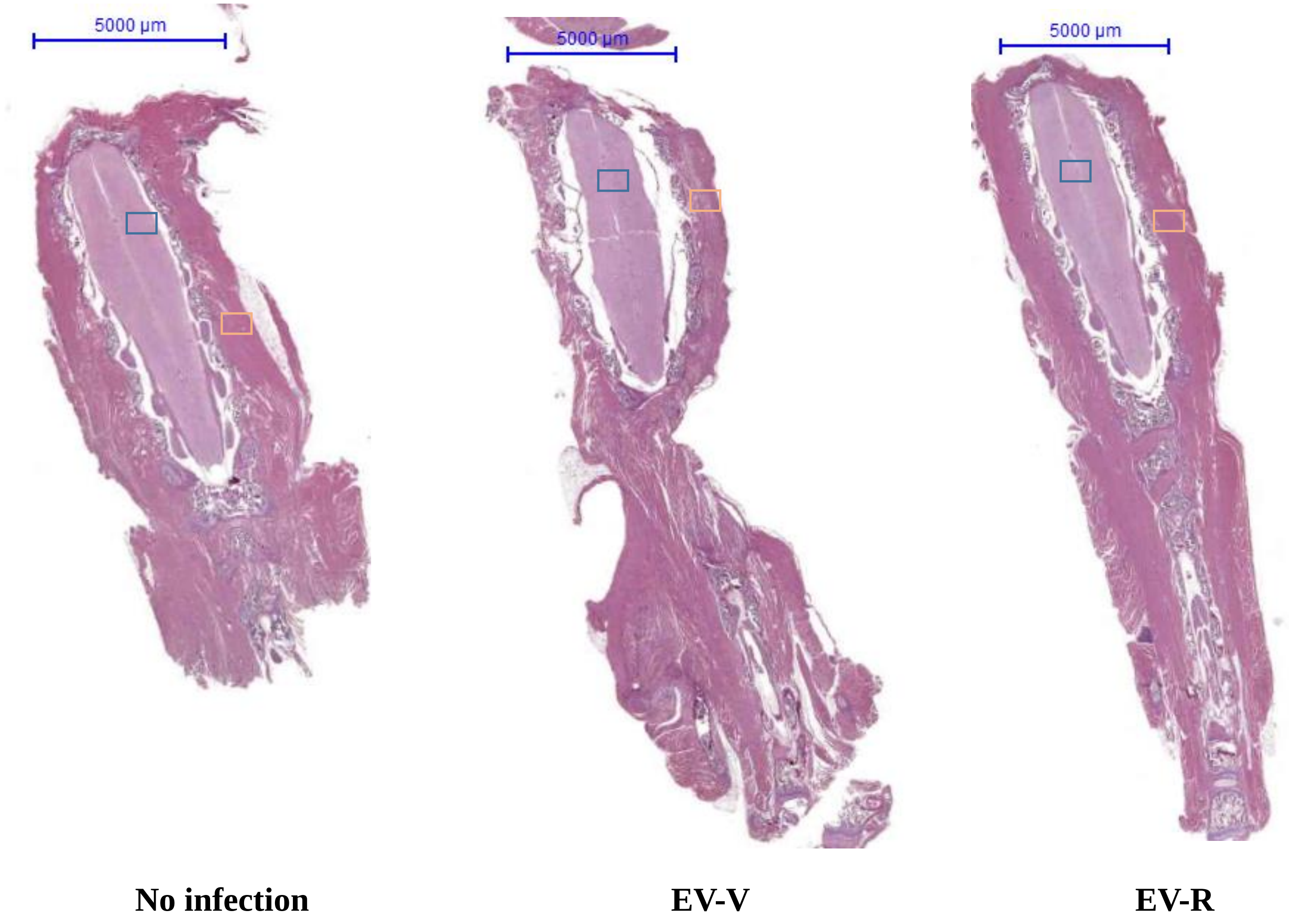

Supplementary Figure S1. Isolation of the spine tissues from EV71-infected hSCARB2-Tg mice for histochemistry.
Whole spines from individual naïve and hSCARB2-Tg mice challenged with $5{\times}10^6$ pfu of EV-V or EV-R were isolated on day 4 pi. After sectioning, the spinal cord and surrounding muscle were consistently marked with grids for staining with dyes or antibodies as described in the Materials and Methods. The stained images are shown at 4x magnification. A scale bar of 5000 μm is indicated in the field.

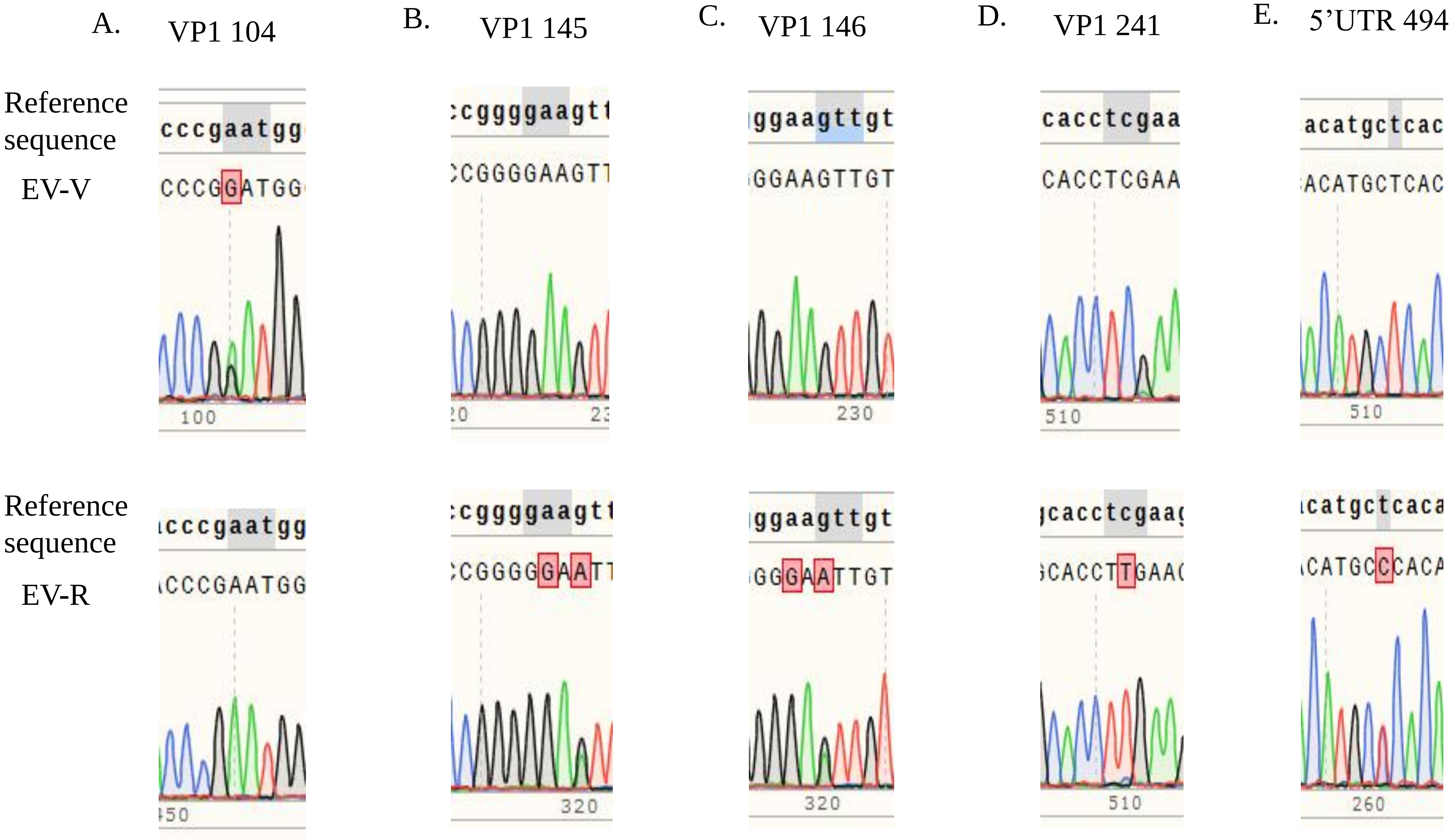

Supplementary Figure S2. Alignment of the EV-V and EV-R nucleotide sequences.

The whole genomes of EV-V and EV-R were sequenced as described in the Materials and Methods. The graph shows the mutation sites VP1-104, 145, 146, 241, and 5'-UTR 494 from EV-V and EV-R. The respective reference sequences (GenBank: AF304457.1) are also included.
